# Extracellular volume quantification by dynamic equilibrium cardiac computed tomography in cardiac amyloidosis

**DOI:** 10.1016/j.jcct.2015.07.001

**Published:** 2015

**Authors:** Thomas A. Treibel, Steve Bandula, Marianna Fontana, Steven K. White, Janet A. Gilbertson, Anna S. Herrey, Julian D. Gillmore, Shonit Punwani, Philip N. Hawkins, Stuart A. Taylor, James C. Moon

**Affiliations:** a The Heart Hospital, 16-18 Westmoreland Street, London, W1G 8PH, UK; b Institute of Cardiovascular Science, University College London, London, WC1E 6BT, UK; c Centre for Amyloidosis and Acute Phase Proteins and National Amyloidosis Centre, Royal Free Campus, University College London, Rowland Hill Street, London, NW3 2PF, UK; d Centre for Medical Imaging, University College London, 250 Euston Road, London, NW1 2PG, UK

**Keywords:** Extracellular space, Cardiac imaging techniques, CMR, CCT, Amyloidosis, EQ-CMR, Equilibrium-infusion contrast Cardiovascular Magnetic Resonance, DynEQ-CT, Bolus-only Dynamic-Equilibrium Computed Tomography, ATTR amyloidosis, Transthyretin amyloidosis, AL amyloidosis, Immunoglobulin light-chain amyloidosis, DPD, 3,3-diphosphono-1,2-propanodicarboxylicacid, ECV, Extracellular Volume fraction, AS, Aortic Stenosis, ShMOLLI, Shortened Modified Look-Locker Inversion Recovery, TTR, Transthyretin protein, LGE, Late gadolinium enhancement, LVH, Left ventricular hypertrophy, LV, Left ventricle, RV, Right ventricle

## Abstract

**Background:**

Cardiac involvement determines outcome in patients with systemic amyloidosis. There is major unmet need for quantification of cardiac amyloid burden, which is currently only met in part through semi-quantitative bone scintigraphy or Cardiovascular Magnetic Resonance (CMR), which measures ECV_CMR_. Other accessible tests are needed.

**Objectives:**

To develop cardiac computed tomography to diagnose and quantify cardiac amyloidosis by measuring the myocardial Extracellular Volume, ECV_CT_.

**Methods:**

Twenty-six patients (21 male, 64 ± 14 years) with a biopsy-proven systemic amyloidosis (ATTR n = 18; AL n = 8) were compared with twenty-seven patients (19 male, 68 ± 8 years) with severe aortic stenosis (AS). All patients had undergone echocardiography, bone scintigraphy, NT-pro-BNP measurement and EQ-CMR. Dynamic Equilibrium CT (DynEQ-CT) was performed using a prospectively gated cardiac scan prior to and after (5 and 15 minutes) a standard Iodixanol (1 ml/kg) bolus to measure ECV_CT_. ECV_CT_ was compared to the reference ECV_CMR_ and conventional amyloid measures: bone scintigraphy and clinical markers of cardiac amyloid severity (NT-pro-BNP, Troponin, LVEF, LV mass, LA and RA area).

**Results:**

ECV_CT_ and ECV_CMR_ results were well correlated (r^2^ = 0.85 vs r^2^ = 0.74 for 5 and 15 minutes post bolus respectively). ECV_CT_ was higher in amyloidosis than AS (0.54 ± 0.11 vs 0.28 ± 0.04, *p*<*0*.*001*) with no overlap. ECV_CT_ tracked clinical markers of cardiac amyloid severity (NT-pro-BNP, Troponin, LVEF, LV mass, LA and RA area), and bone scintigraphy amyloid burden *(p*<*0*.*001)*.

**Conclusion:**

Dynamic Equilibrium CT, a 5 minute contrast-enhanced gated cardiac CT, has potential for non-invasive diagnosis and quantification of cardiac amyloidosis.

## Highlights

• Cardiac involvement determines outcome in amyloidosis.
• There is major unmet need for quantification of cardiac amyloid burden.
• Dynamic Equilibrium CT (DynEQ-CT) is a 5-minute contrast-enhanced CT scan.
• DynEQ-CT measures the extracellular volume fraction, which increases in amyloid.
• DynEQ-CT has major potential for diagnosis and quantification of cardiac amyloidosis.

## Introduction

Systemic amyloidosis is a multisystem disease caused by the deposition of misfolded fibrillar protein in tissues and organs causing expansion of the extracellular space and impairment of function.^1^, ^2^ Cardiac involvement is a leading cause of morbidity and mortality, with two types commonly infiltrating the ventricular myocardium: immunoglobulin light-chain (AL) type and transthyretin (ATTR) type.^3^, ^4^, ^5^ With current or pending new treatment options for both,^6^, ^7^ improved diagnosis of cardiac involvement is becoming increasingly important. However current clinical algorithms do not allow quantification of cardiac burden.^5^, ^8^, ^9^ Invasive cardiac biopsy is prone to sampling error and unsuitable for monitoring therapy. Although certain types of bone scintigraphy^10^, ^11^ and conventional cardiovascular magnetic resonance (CMR) late gadolinium enhancement (LGE)^12^ can detect cardiac amyloid, neither is truly quantitative and both have limitations – for example, bone tracers are insensitive in AL amyloidosis and SAP scintigraphy does not image cardiac amyloid.

Extracellular volume fraction (ECV) quantified by Equilibrium Contrast CMR (EQ-CMR) tracks amyloid burden.^13^ Equilibrium Computed Tomography can also quantify the ECV from pre and post contrast measurements of attenuation, and shows good correlation with ECV measured by EQ-CMR as well as histological measures of myocardial fibrosis.^14^, ^15^, ^16^ CT has advantages over CMR: ubiquitously available equipment, higher spatial resolution and a simple linear relationship between attenuation (Hounsfield units) and iodine concentration as opposed to the nonlinear effect on relaxivity of hydrogen following administration of gadolinium.

This study was designed to simplify the progenitor equilibrium methods by using a bolus-only, dynamic equilibrium approach (DynEQ-CT) and apply it to an important clinical problem: the non-invasive diagnosis and quantification of cardiac amyloid burden. Our objectives were three-fold: firstly, to evaluate optimal post bolus contrast timing for DynEQ-CT; secondly, to assess the ability to quantify myocardial amyloid against conventional semi-quantitative methods (bone scintingraphy) and clinical markers of cardiac dysfunction; and thirdly, to compare ECV_CT_ against ECV_CMR_.

## Material and methods

All research was carried out at University College London Hospital and the Royal Free NHS Trusts, London, UK, between January 2013 and December 2013. The study was approved by the ethical committee of U.K. National Research Ethics Service (REC reference 09/H0716/75) and conformed to the principles of the Helsinki Declaration. All subjects gave written informed consent to participate in the study. Exclusion criteria were uncontrolled arrhythmia, impaired renal function (estimated glomerular filtration rate <60 mL/min), or contraindications to MR imaging (e.g. implanted devices – exclusion from CMR only). A total of 53 patients were recruited: 26 with systemic amyloidosis (ATTR and AL) and 27 comparator controls with aortic stenosis. All patients underwent 12-lead ECG, DynEQ-CT, EQ-CMR, echocardiography for diastolic function assessment according to European Society of Echocardiography criteria,^17^ assays of NT-proBNP and 6-minute walk test where health and patient choice permitted (e.g. arthritis, postural hypotension, neuropathy). Troponin T was measured in amyloid patients only. The ATTR group also underwent bone scintigraphy using 3,3-diphosphono-1,2-propanodicarboxylicacid (DPD).^10^

### Amyloid patients

26 patients with systemic amyloidosis were recruited. Eighteen patients had ATTR amyloidosis (16 male, age 68 ± 8 years), and eight patients (5 male, age 56 ± 12 years) had systemic AL amyloidosis. For ATTR, cardiac amyloidosis was defined by presence of ATTR amyloid in a myocardial biopsy (Congo red and immunohistochemical staining) or positive DPD scintigraphy; 6 TTR patients had positive myocardial biopsies.^10^ Definite cardiac involvement by DPD was defined as DPD grade 2 or 3, and possible cardiac involvement as grade 1 (i.e. minimal cardiac DPD uptake) in the absence of LVH; in practice, none of these patients had LVH. All TTR patients also underwent sequencing of exons 2, 3, and 4 of the TTR gene: 14 had senile systemic amyloid (SSA) and four TTR patients were familial (V122I [n = 1], V30M [n = 1], E54G [n = 2]). For AL, systemic AL amyloidosis was proven with biopsies from non-cardiac tissues. Cardiac categorization was based on international consensus criteria^18^ but with an additional “possible involvement” category, as previously described.^19^ Overall, 21 patients were categorized as having definite cardiac amyloidosis (14 ATTR; 7 AL) and 5 patients (4 ATTR; 1 AL) as having possible cardiac involvement.

### Comparator group

Twenty-seven age- and sex-matched patients with severe aortic stenosis (19 male, age 68 ± 8 years) underwent DynEQ-CT and CMR (n = 22 with 5 exclusions due to claustrophobia or pacemaker). AS was chosen as a comparator for having LV remodeling and hypertrophy, and allowing for cardiac amyloid to be excluded histologically on myocardial biopsy (Congo red staining) taken during aortic valve surgery as part of a separate study.

### DynEQ-CT Protocol

**Fig. 1:**
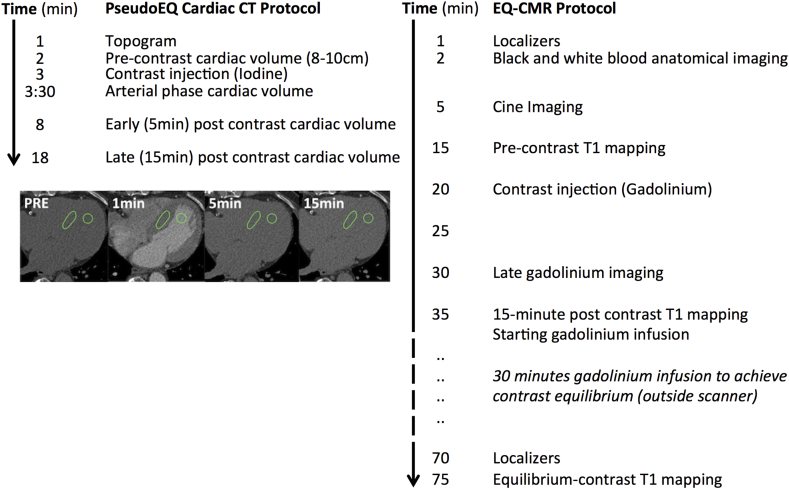
PseudoEQ Cardiac CT and EQ-CMR Protocols: EQ-CMR was performed either after or at least 24 hours prior to the CT to avoid residual gadolinium causing an increase in measured attenuation. The CMR protocol for amyloidosis is 3.5 × longer than the CT protocol.

The DynEQ-CT protocol consisted of three steps (Fig. 1 for flow chart): first, a CT scan to obtain baseline pre-contrast blood and myocardial attenuation in Hounsfield units (HU); second, contrast administration and delay so the contrast distributes into a blood:myocardial dynamic equilibration; third, a repeat scan to re-measure blood and myocardial attenuation. The ratio of the change in blood and myocardial attenuation (ΔHU) represents the contrast agent partition coefficient. If the blood volume of distribution is substituted in (1 minus venous hematocrit; obtained prior to imaging), the myocardial extracellular volume, ECV_CT_, is obtained, reflecting the myocardial interstitium: ECV_CT_ = (1−Hematocrit) × (ΔHU_tissue_/ΔHU_blood_).

CT examinations were performed on a 64–detector row CT scanner (Somatom Sensation 64; Siemens Medical Solutions, Erlangen, Germany). A topogram was used to plan CT volumes from the level of the aortic valve to the inferior aspect of the heart, typically a 10 cm slab. Cardiac scans (tube voltage, 120 kV; tube current–time product, 160 mAs; section collimation, 64 detector rows, 1.2-mm section thickness; gantry rotation time, 330 msec) were acquired with prospective gating (65%–75% of R-R interval), and reconstructed into 3-mm-thick axial sections with a B20f kernel.

To establish the best timing, post contrast imaging was performed at both 5- and 15-minutes following a bolus of Iodixanol (652 mg/mL) at a standard dose of 1 mL/kg and injection rate of 3 ml/sec without a saline chaser. An additional single 3 mm slice acquisition at 1-minute (other parameters as previously described) was introduced in the amyloid cohort to aid blood:myocardial boundary detection for segmentation of the myocardium during analysis.

**Fig. 2:**
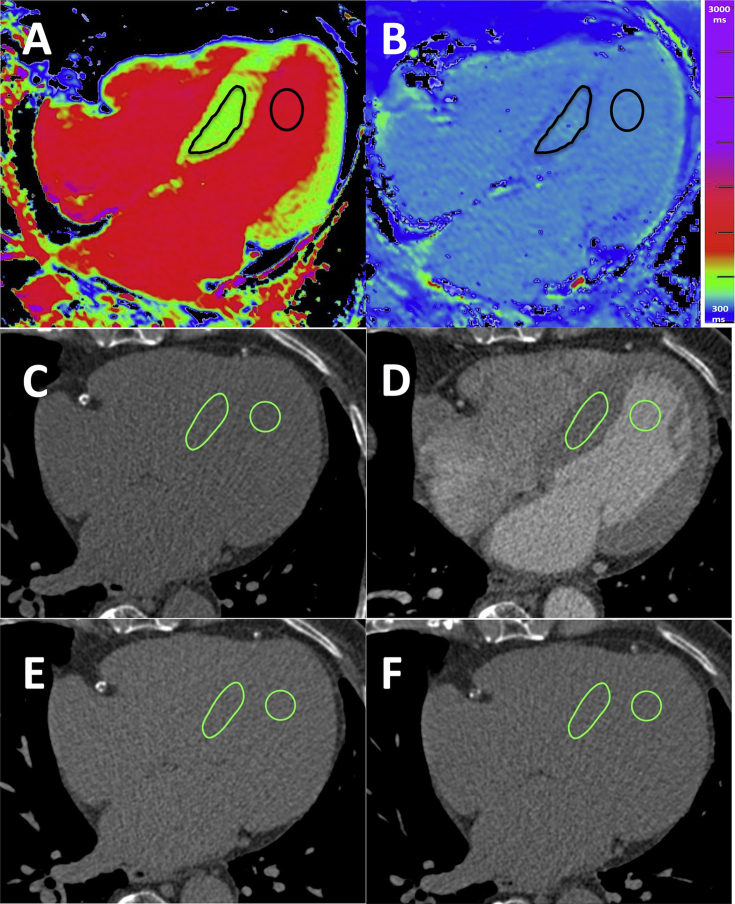
Examples of typical CMR and CT analysis: Top row displays regions of interest (ROIs) in CMR T1 maps images acquired before (A) and after gadolinium contrast (B). Middle and bottom rows show ROIs in gated cardiac CT images acquired pre-contrast (C), 1 minute (D), 5 and 15 minutes post iodine contrast (E + F). ROIs were drawn in the myocardial septum and blood pool.

CT image analysis was performed using a free and open-source Digital Imaging and Communications in Medicine viewer (OsiriX v4.1.2; Pixmeo, Bernex, Switzerland) independently by two experienced readers blinded to all other study data; this was repeated by the second reader to establish inter- and intra-observer agreement. Regions of interest (ROIs) were drawn in the contrast-enhanced 1-minute acquisition in axial sections and propagated to the pre-contrast, 5-minute and 15-minute acquisitions. For myocardium, polygonal ROIs were drawn in an axial slice containing the greatest area of myocardial septum; for the blood pool, circular ROIs were drawn in the LV blood pool away from papillary muscles and the myocardial septum to avoid the endocardial edge and therefore partial voluming (Fig. 2). Myocardial and blood attenuation values were used to calculate the ECV fraction as described.

Signal-to-noise ratios (SNR) were measured in five myocardial ROIs per time point from the ratio of the average HU attenuation value to the standard deviation of the HU attenuation. Radiation exposure was quantified using the dose-length product multiplied by a chest conversion coefficient (κ = 0.014 mSv/mGy cm).^20^

### EQ-CMR protocol

EQ-CMR was performed using a 1.5-T scanner (Avanto; Siemens Medical Imaging, Erlangen, Germany) either after or at least 24 hours prior to the CT to avoid residual gadolinium causing an increase in measured attenuation. In addition to the standard CMR protocol (Fig. 1 for flow chart), T1 mapping for ECV_CMR_ quantification was performed using ShMOLLI (Shortened Modified Look-Locker Inversion recovery),^21^ providing a single-section T1 map in one breath-hold.^22^ For CMR image analysis, a large ROI was drawn manually by a reader blinded to the clinical data on each image to deﬁne the septum and blood pool; source data and error maps were used for quality control (Fig. 2).^23^

### 99mTc-DPD bone scintigraphy

For the ATTR patients, bone scintigraphy as a semiquantitative test of myocardial amyloid burden was performed as previously described.^10^, ^11^ Cardiac retention of 99mTc-DPD was visually scored as: Grade 0 – no myocardial uptake; Grade 1 – mild cardiac uptake with no significant attenuation of normal bony uptake; Grade 2 – moderate cardiac uptake with some attenuation of the bone signal; Grade 3 – strong cardiac uptake with little or no bone uptake.

### Statistical analysis

A statistical package (SPSS, version 22) was used for all data analysis. Continuous variables were expressed as mean ± SD and non-parametric variables as median with inter-quartile. The association between ECV_CT_ and ECV_CMR_ was assessed by using Pearson correlation; normal distribution was assessed by using the Shapiro Wilkinson test. Agreement between ECV_CT_ and ECV_CMR_ was assessed by Bland-Altman comparison. A *p value* <*0*.*05* was considered statistically significant.

## Results

Baseline characteristics of twenty-six systemic amyloidosis and twenty-seven AS patients are shown in Table 1. Compared to AS, patients with amyloidosis had a significantly higher LV mass (116 ± 40 vs 103 ± 27 g/m^2^; *p* = *0*.*05*), higher indexed left atrial area (15.3 ± 3.4 vs 13.2 ± 3.7 cm^2^/m^2^; *p* = *0*.*05*), lower LVEF (59 ± 15 vs 69 ± 13%; *p* = *0*.*02*), lower indexed stroke volume (42 ± 10 vs 50 ± 13 ml/m^2^; *p* = *0*.*04*), worse diastolic function (E/A ratio: 1.46 ± 0.94 vs 0.93 ± 0.55 ml/m^2^; *p* = *0*.*02*) and shorter distance on 6-minute walk test (356 ± 136 vs 469 ± 168 m; *p* = *0*.*01*). There was no significant between group difference in systolic or diastolic blood pressure, NT-proBNP or Troponin.

### Technical development: DynEQ-CT ECV performance at 5 and 15 minutes

**Fig. 3:**
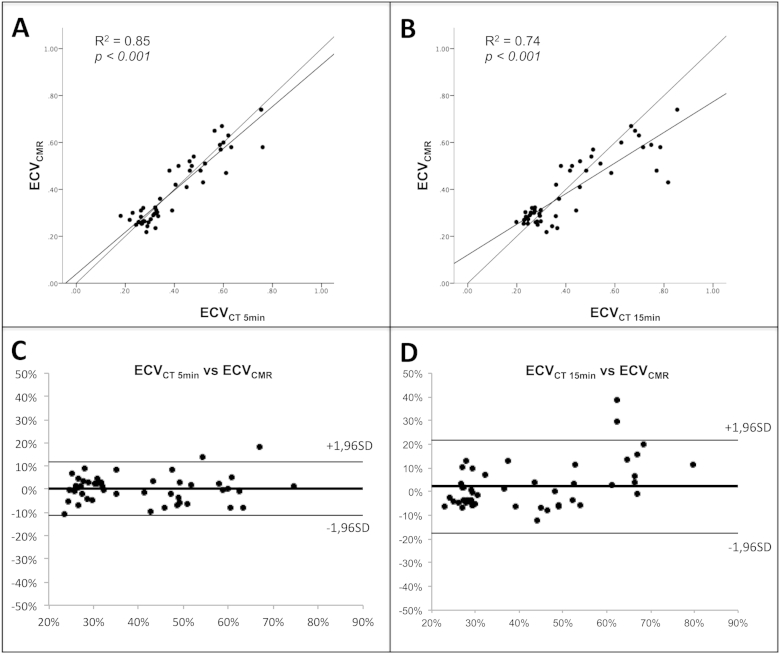
Correlation and agreement of ECV derived by CT and CMR: Top row show ECV_CMR_ and ECV_CT_ correlations; the 5 minute CT (A) correlates better than at 15 minutes (B) (r^2^ = 0.85 vs r^2^ = 0.74; *p*<*0*.*001*). Bottom row shows Bland-Altman comparisons of the ECV measurement by CMR versus CT at 5 minutes (C) and 15 minutes (D). ECV differences are expressed as a percentage, calculated by subtracting ECV_CT_ from ECV_CMR_) against mean ECV (solid thick line), with lower (bottom thin line) and upper (top thin line) 95% limits of agreement.

Comparison of the signal to noise ratios showed that the SNR was significantly higher in the 5 minute rather than 15 minute scan (4.7 ± 0.9 vs 3.9 ± 0.9, *p*<*0*.*001*). ECV_CT_ at 5 minutes was strongly correlated with ECV_CMR_ (r^2^ = 0.85, *p*<*0*.*001*) but correlation was weaker at 15 minutes (r^2^ = 0.74, *p*<*0*.*001*; Fig. 3). Bland-Altman comparisons of ECV_CT_ and ECV_CMR_ demonstrated no bias at 5 minutes post contrast, with slight bias at 15 minutes (4%, no slope). Compared to the 5 minute time point, the 95% confidence limits were nearly twice as wide at 15 minutes (95% confidence limits: −18%, 22% vs −11%, 11%).

The mean total calculated effective radiation dose for the whole DynEQ-CT protocol (including both post contrast time points) was 1.56 mSv ± 0.58 mSv, with a mean administered total Iodixanol (Visipaque) volume of 78 mL ± 11 mL.

For DynEQ-CT, inter- and intra-observer agreement was excellent for myocardial (ICC = 0.92 and ICC = 0.94, respectively) and blood pool (ICC = 0.96 and ICC = 0.99, respectively) attenuation measurements. Similarly for ECV, excellent agreement was found (ICC = 0.95 and ICC = 0.98, respectively).

### Diagnosis of cardiac amyloid by ECV

**Fig. 4:**
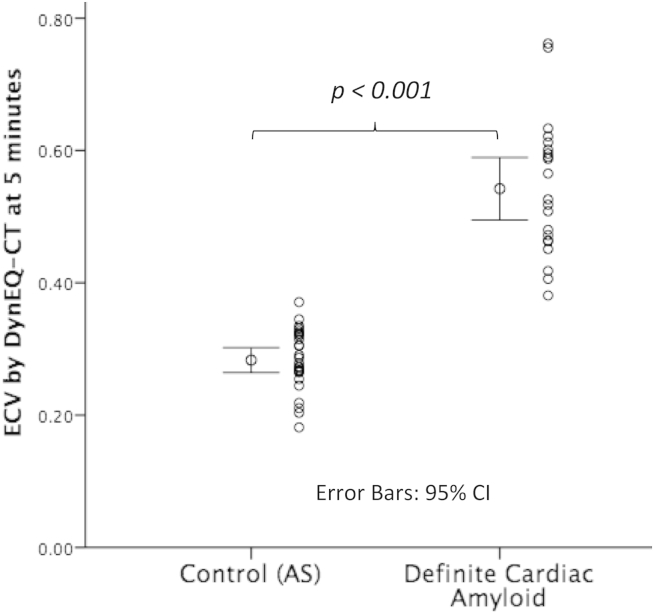
ECV in patients with definite cardiac amyloidosis: Myocardial ECV by DynEQ-CT at 5 minutes was higher in all patients with definitive cardiac amyloidosis than in patients with severe aortic stenosis (0.54 ± 0.11 vs 0.28 ± 0.04, *p < 0.001*).

ECV_CT_ was significantly higher in amyloid patients with definitive cardiac involvement than aortic stenosis (0.54 ± 0.11 versus 0.28 ± 0.04, *p*<*0*.*001*; Fig. 4). Like ECV_CMR_, ECV_CT_ discriminated 100% between amyloid patients with definitive cardiac involvement and patients with aortic stenosis (ie there was no overlap in ECV between the two cohorts). Furthermore, ECV_CT_ showed a higher ECV in ATTR than AL amyloidosis (0.56 ± 0.11 versus 0.43 ± 0.12, *p* = *0*.*03*), but with wide overlap between groups.

### ECV tracks clinical parameters

**Fig. 5:**
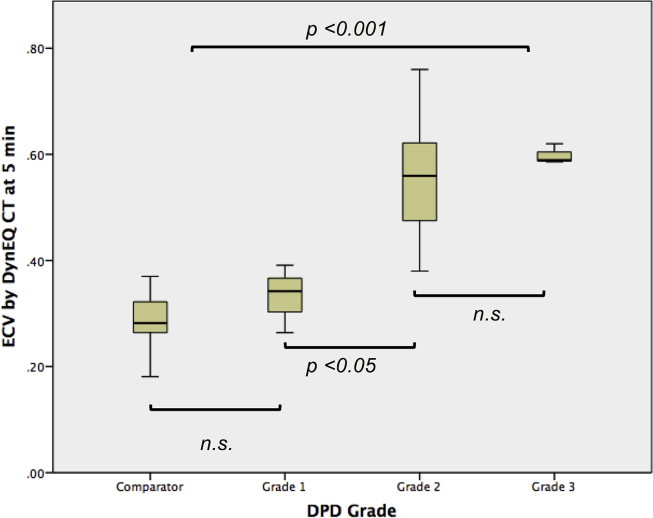
ECV tracks amyloid burden measured by DPD bone scintigraphy: ECV vs DPD grade in 26 patients with systemic amyloidosis (27 patients with aortic stenosis used as comparator – no evidence of cardiac involvement on myocardial biopsy).

ECV_CT_ tracked clinical parameters as equally well as CMR, with DynEQ-CT ECV at 5 minutes consistently better than at 15 minutes (Table 2): for the 5 minute results, ECV_CT_ increased with increasing index LV mass (r = 0.43, *p* = *0*.*003*), increasing indexed LAA (r = 0.49, *p* = *0*.*001*), lower LV ejection fraction (r = −0.45, *p* = *0*.*002*), worse LV diastolic function (E/A ratio: r = 0.51, *p*<*0*.*001*), reduced distance on 6MWT (r = 0.35, *p* = *0*.*02*), increasing NT-pro-BNP levels (r = 0.66, *p* = *0*.*001*) and Troponin T levels (r = 0.49, *p* = *0*.*03*). In ATTR amyloid, ECV_CT_ tracked amyloid burden semi-quantitatively measured by DPD bone scintigraphy (*p*<*0*.*001*) (Fig. 5).

## Discussion

This study showed that measuring the myocardial extracellular volume, ECV_CT_, using a simple 5-minute, gated cardiac CT protocol, DynEQ-CT, can distinguish cardiac amyloidosis from another disease with myocardial hypertrophy, aortic stenosis. ECV_CT_ is a quantitative parameter and compares well with the previously validated standard of ECV_CMR_ but is easier and quicker to measure. ECV_CT_ tracked cardiac amyloid burden, which is a major marker of outcome in systemic amyloidosis. This protocol can simply be added to routine CT coronary angiography protocols with only a small increase in radiation dose. It has the potential to offer a unique insight into the myocardial interstitium and provide a new diagnostic test for an under-diagnosed and now treatable cause of LVH and heart failure: cardiac amyloidosis.

Our previous protocol using a primed infusion to reach contrast equilibrium was logistically cumbersome and time consuming, therefore development of a bolus-only approach brings ECV, this promising novel biomarker, a step closer to routine clinical applicability. In our paper we show disease-specific utility of the CT approach. Furthermore, we unequivocally demonstrated that imaging at 5 minutes post bolus was superior to acquisition at 15 minutes, with superior SNR, correlation to the ECV_CMR_, correlation to clinical parameters known to track cardiac disease, and to semiquantitative bone tracer scanning results in TTR amyloid. The theoretical advantage of allowing equilibration of the iodine concentration in myocardium and blood between the 5 minute and 15 minute scan is out-weighed by the loss in signal due to overall lower iodine concentrations. Peculiar to cardiac amyloid, as the ECV is so high, early post contrast images may not allow easy identification of the boundary between myocardium and blood. This is due to the myocardial ECV being as high or even higher than that of the plasma such that pre and post contrast attenuation are very similar. The addition of a low dose, early arterial phase scan facilitated blood myocardial segmentation overall. The resulting triple phase scan using a 64 slice scanner had a mean overall radiation dose of 1.5 mSv – significantly lower than conventionally used bone tracer scintigraphy (4 mSv).

Comparison to previous CT_ECV_ work is limited to two studies by Nacif and colleagues comparing heart failure patients with controls: In the first study, subjects underwent CMR and CCT with CT_ECV_ and yielding results in line with the CMR ECV literature and similar to our AS cohort (28.6 ± 4.4% vs 31.6 ± 5.1%, *p* = *0*.*03*).^15^ In the second study, whole heart 3D ECV was quantified; ECV was substantially higher than in the previous CT study by the same group and in CMR_ECV_ studies (41 ± 6%, 33 ± 2%; *p* = *0*.*02*),^16^ raising the concern that the partial voluming of the blood pool may have occurred. In this context, our CT ECV results in confirmed cardiac amyloidosis were 6 standard deviations higher than in the control subjects.

From a diagnostic perspective, ECV_CT_ was higher in patients with definite cardiac amyloidosis than the comparator group with LVH due to AS with no overlap, which mirrored previous findings by ECV_CMR_. Furthermore, ECV_CT_ correlated with established markers of cardiac amyloid severity using structural and functional (LVEF and SV) parameters, biomarkers, and function capacity as well as cardiac amyloid burden measured semi-quantitatively by bone tracer scintigraphy. Although the role of cellular hypertrophy and diffuse fibrosis is unknown in cardiac amyloid, the ECV expansion seen with amyloid deposition is ten times larger than that seen with diffuse fibrosis in aortic stenosis, hypertension or hypertrophic cardiomyopathy.^24^, ^25^, ^26^

The ECV_CT_ technique carries some advantages in clinical practice; CMR is not suitable in around 10% of patients (due to claustrophobia or many cardiac pacemakers). Furthermore, there are concerns that the CMR signal is affected by limits to the fast exchange of protons (this does not apply to CT).^23^ The CT approach is cheaper, completed in 5 minutes, and widely available, providing high-resolution 3D ECV volumes with two single breath-hold acquisitions, and the scanner design can accommodate patients with obesity and claustrophobia. Although it has a lower signal to noise ratio, and probably more dependency on a reasonable eGFR, ECV_CT_ therefore has several advantages over ECV_CMR_.

### Limitations and future steps

In this study, the DynEQ-CT technique was compared to the best, currently available non-invasive techniques for the quantification of ECV (EQ-CMR) and TTR amyloid burden (DPD scintigraphy), but no invasive biopsy data was available for this validation. However, with the patchy distribution of cardiac amyloid deposits, biopsy data may not be superior. DynEQ-CT and EQ-CMR only identify general cardiac amyloid deposition (which is important to trigger therapy); tissue diagnosis is still necessary, but can be achieved from more accessible, non-cardiac tissue in this systemic disease. The data presented here is from a small study in a single specialized center, aimed to be hypothesis generating. A larger study is required to test these, develop this test further, and compare the head-to-head diagnostic performance of CT and CMR. The 64-slice-CT-system employed here may reflect the most commonly available systems available, but does not take advantage of newer systems that offer iterative reconstruction algorithms, dual energy acquisition and larger detector arrays that allow acquisition of whole heart, isotropic volumes of in one heart beat and at low radiation dose. In conjunction with 3D image registration and processing, this allows creation of whole heart ECV maps.^16^ Given the exposure to ionizing radiation, patients with severe aortic stenosis were deemed as adequate control cohort, avoiding exposure of healthy volunteers. Furthermore, AS patients were age-/gender-matched and had ventricular hypertrophy. As a research project, we limited the study to subjects with an eGFR of >60, a significant limitation for AL amyloid where 50% of patients with significant amyloid have renal impairment.

## Conclusion

DynEQ-CT is a simple 5-minute, gated, easily implementable contrast-enhanced cardiac CT scan that can quantify cardiac amyloid burden, track disease severity and appears to be as effective as cardiovascular MR. Advantages over cardiovascular MR include scanner availability and cost, reduced examination time and applicability to those with CMR contraindications.

## Disclosures

All authors have reported that they have no relationships relevant to the contents of this paper to disclose.

## Source of funding

T.A.T and S.B. are supported by Doctoral Research Fellowships from the NIHR, UK (NIHR-DRF- 2013-06-102/NIHR-DRF- 2011-04-008). M.F and S.K.W. are supported by Clinical Research Training Fellowships from the British Heart Foundation (grants FS/12/56/29723 and FS/10/72/28568). J.C.M. has received grant funding from GlaxoSmithKline. Stuart Taylor is an NIHR senior investigator. This work was undertaken at University College London Hospital, which received a proportion of funding from the UK Department of Health National Institute for Health Research Biomedical Research Centers funding scheme.

## Figures and Tables

**Table 1:** Baseline characteristics of amyloidosis and aortic stenosis patients.

	Systemic Amyloidosis	Comparator with Aortic Stenosis	*p*-*value*
N	26	27	
Men/women	21/5	19/8	
Age, yrs	64 ± 14	68 ± 8	*0*.*2*
eGFR, ml/min/1.73 m^2^	71 ± 11	78 ± 19	*0*.*1*
LV structure by CMR			
Indexed LV mass, g/m^2^	116 ± 40	103 ± 27	*0*.*05*
Indexed LA area, cm^2^/m^2^	15.3 ± 3.4	13.2 ± 3.7	*0*.*05*
LV systolic function by CMR			
LVEF, %	59 ± 15	69 ± 13	*0*.*02*
Indexed SV, ml/m^2^	42 ± 10	50 ± 13	*0*.*02*
Echocardiography			
E-wave	0.86 ± 0.20	0.73 ± 0.32	*0*.*08*
E/A	1.46 ± 0.94	0.93 ± 0.55	*0*.*02*
E/E′	14.7 ± 7.2	13 ± 8	*0*.*1*
E-deceleration time, ms	178 ± 54	246 ± 76	*0*.*001*
Aortic Valve Peak Gradient	7 ± 1	68 ± 21	*0*.*001*
Clinical Parameters			
6 minutes walking test, meters	356 ± 130	469 ± 168	*0*.*01*
SBP (mmHg)	129 ± 22	131 ± 18	*0*.*5*
DBP (mmHg)	76 ± 12	74 ± 11	*0*.*3*
Atrial Fibrillation	3 (11.5%)	2 (8%)	
Biomarkers			
NT-proBNP, pmol/L	356 (24-1426)	155 (8-568)	*0*.*04*
Troponin T, pmol/L	0.080 (0.01-0.24)	NA	

Values are mean ± SD or %.

Patients with systemic amyloidosis encompassing light-chain and transthyretin amyloidosis and aortic stenosis patients with severe stenosis awaiting valve replacement.

eGFR, estimated glomerular filtration rate; NT-proBNP, N-terminal pro-brain natriuretic peptide; CMR, cardiovascular magnetic resonance; EDV, end diastolic volume; ESV, end systolic volume; LVEF, left ventricular ejection fraction; SV, stroke volume; LV, left ventricular; LAA, left atrial area.

**Table 2:** Correlations between ECV_CT_ and ECV_CMR_ with clinical parameters.

	ECV_CMR_ (R)	ECV_CT_ 5-minutes (R)	ECV_CT_ 15-minutes (R)
LV structure by CMR			
Indexed LV mass, g/m^2^	0.40‡	0.43‡	0.44‡
Indexed LA area,cm^2^/m^2^	0.45‡	0.49‡	0.45†
LV systolic function by CMR			
LVEF, %	−0.46†	−0.43†	−0.38†
Indexed SV,ml/m2	−0.34†	−0.28 ns	−0.25 ns
LV diastolic function by echo			
E	0.47‡	0.35†	0.24 ns
E/A	0.52‡	0.51‡	0.43‡
E/E′	0.48‡	0.49‡	0.47‡
E-deceleration time, ms	−0.50‡	−0.45‡	−0.40‡
Clinical Parameters			
SBP (mmHg)	−0.43†	−0.26 ns	−0.35†
DBP (mmHg)	−0.15 ns	−0.08 ns	−0.06 ns
6 minutes walking test, meters	−0.39‡	−0.35†	−0.24 ns
Biomarkers			
NT-proBNP, pmol/L	0.59‡	0.64‡	0.45†
Troponin T, pmol/L	0.50†	0.49†	0.0.6 ns

‡P < 0.01 level; †P < 0.05; ns non-significant.

R = Pearson correlation coefficient; CMR, cardiovascular magnetic resonance; CT, computed tomography; ECV, extracellular volume fraction; NT-proBNP, N-terminal pro-brain natriuretic peptide; EDV, end diastolic volume; ESV, end systolic volume; LVEF, left ventricular ejection fraction; SV, stroke volume; LV, left ventricular; LAA, left atrial area.
